# Availability of treatment resources for the management of acute toxic exposures and poisonings in emergency departments among various types of hospitals in Palestine: a cross-sectional study

**DOI:** 10.1186/1757-7241-22-13

**Published:** 2014-02-21

**Authors:** Sa’ed H Zyoud, Samah W Al-Jabi, Yara I Bali, Afnan M Al-Sayed, Waleed M Sweileh, Rahmat Awang

**Affiliations:** 1 Poison Control and Drug Information Center (PCDIC), College of Medicine and Health Sciences, An-Najah National University, Nablus, Palestine; 2 Department of Pharmacology and Toxicology, College of Medicine and Health Sciences, An-Najah National University, Nablus, Palestine; 3 WHO Collaborating Centre for Drug Information, National Poison Centre, Universiti Sains Malaysia (USM), Penang, Malaysia; 4 Department of Clinical and Comunity Pharmacy, College of Medicine and Health Sciences, An-Najah National University, Nablus, Palestine; 5 PharmD Program, College of Medicine and Health Sciences, An-Najah National University, Nablus, Palestine

**Keywords:** Availability, Treatment resources, Emergency departments, Poisoning, Hospitals, Palestine

## Abstract

**Background:**

Poisoning exposures continue to be a significant cause of morbidity and mortality worldwide. The lack of facilities, treatment resources, and antidotes in hospitals may affect the treatments provided and outcomes. This study aimed to determine the availability of gastrointestinal (GI) decontamination, stabilisation, elimination enhancement resources, and antidotes for the management of acute toxic exposures and poisonings in emergency departments (EDs) among various types of governmental and private hospitals in Palestine.

**Methods:**

A cross-sectional study using semi-structured questionnaire was performed. Data were collected based on hospital resources; GI decontamination, stabilisation, elimination enhancement resources and antidotes from Palestinian hospitals.

**Results:**

Eighteen hospitals (94.7%) have responded. Among them, paracetamol poisoning was the most frequently reported cases by EDs (mean frequency score = 7.6 ± 2.1), followed by bee stings (mean = 6.9 ± 2.7) and organophosphate poisoning (mean = 6.7 ± 2.7). The availabilities of most resources related to GI decontamination items varied substantially with hospital type, but these differences were not statistical significant. The availability of stabilisation resources was not significantly different between hospitals types. For the availability of techniques used to enhance the elimination of toxic substances, there were variations between the hospitals types. However, these differences were not statistical significant, except for haemodialysis (p = 0.003) which was more available in governmental hospitals. For the availability of antidotes, none of the hospitals had sufficient stock of all antidotes listed. In relation to hospital type, there was variability in the availability of antidotes, but this did not reach statistical significance, except for deferoxamine (p < 0.001), which was available in all governmental hospitals but none of the private hospitals.

**Conclusions:**

The availability of treatment resources and antidotes in Palestinian hospitals was not adequate except for stabilisation resources. The availability of such resources acts as a marker for the level of readiness of hospital EDs in Palestine for the management of acute toxic exposure and poisoning. The implementation of a minimum list of antidotes and treatment resources would be useful to increase the level of resources. Coordination between Palestinian poison control and drug information centre and hospitals is also important.

## Introduction

With the availability of a vast number of chemicals and drugs, acute poisoning is a medical emergency [1,2] and is considered one of the most common reasons for visiting emergency departments (EDs). Poisoning exposures continue to be a significant cause of morbidity and mortality worldwide [3]. The National Vital Statistics Reports showed that poisoning was the fifth leading cause of injury and death in the United States of America in 2010 [4], while the exact incidence of this problem in Palestine remains uncertain and information available is limited due to under-diagnosis and underreporting. The growing incidence of poisoning has highlighted the importance for countries to have special programs for poison control and, in particular, the facilities for diagnosis, treatment, and prevention of poisoning [5].

If a poisoning is recognised early and appropriate supportive care is initiated rapidly, the majority of patient outcomes will be good [6]. An example of this is the treatment of patients diagnosed with paracetamol poisoning with N-acetylcysteine within 8 hours [7]. In addition, the treatment of poisoning cases in the ED begins with stabilising the patient and assessing the vital signs, starting with ABC (airway, breathing and circulation), which is sometimes followed by gastrointestinal (GI) decontamination or the immediate use of an antidote [6,8]. Supportive measures include the use of commonly stocked medicines such as adrenaline and sodium bicarbonate [9].

Furthermore, since the timely use of antidotes prevents death and shortens the length of hospitalisation, as well as reducing the patient’s pain and suffering, maintaining a sufficient stock of antidotes is the responsibility of any hospital that provides emergency health care [10]. If a poisoned patient needs a certain antidote that is not available in a particular hospital, then the treatment options include supportive measures, borrowing antidotes from another hospital or transferring the patient to the other hospital [9].

To the best of our knowledge, there is a worldwide lack of studies evaluating the preparedness and the availability of the necessary resources to treat poisoning cases. Published research in this area has concentrated mainly on the investigation of antidote availability and preparedness for disaster management. Over the past years, several studies have shown that the unavailability of antidotes is common in health care facilities and sufficient stocking of antidotes remains a problem worldwide [9,11-19]. Also, there are no studies to date that have addressed the level of preparedness of hospital EDs in Palestine for the management of acute toxic exposure and poisonings. Furthermore, only one attempt has been made to identify the availability of antidotes in one district in Palestine [20]. Since poisoning remains a serious problem in Palestine [3,21], and since various facilities are frequently not available, we initiated a countrywide survey to describe the availability of current facilities and the anticipated requirements in Palestine. The purpose of this study was to determine the availability of treatment resources for the management of acute toxic exposures and poisonings in EDs among various types of hospitals in Palestine, and to compare the availability of such facilities among various types of hospitals.

## Methods

### Study design

This was a cross-sectional survey study using semi-structured questionnaire that involved descriptive and comparative analysis.

### Palestinian health system and services

Palestine, also known as the occupied Palestinian territories is located in the Middle East. The key player for the health system and services in Palestine is the Palestinian government through the Palestinian Ministry of Health (PMOH). Other major providers of health care in Palestine include the Palestinian Non-Governmental Organizations (NGOs), the United Nations Relief and Works Agency for Palestine Refugees in the Near East (UNRWA), the Palestinian Military Medical Services (PMMS) and private for profit organizations [22]. The bulk of health services and health expenditure are provided by the PMOH through 458 primary health care centers distributed all through the West Bank and Gaza. The international refugee agency, UNRWA, operates 102 primary health care centers that provide free medical services to Palestinians in refugee camps in the West Bank and Gaza. The NGOs sector operates 206 primary health care centers and general clinics while the PMMS operates 23 primary health care centers and clinics distributed through different districts in West bank and Gaza strip [23]. In the West bank, the PMOH listed 21 public hospitals with EDs, of which 12 were governmental hospitals and 9 were private hospitals.

### Study area and sample size

The data collection was conducted from July to October 2012, and the intention was to cover all hospitals (including government and private hospitals).The primary targets were the EDs from these hospitals that receive poisoning cases, as well as the central pharmacy of each hospital which was responsible for the availability of treatment resources. A list of hospitals was extracted from the Ministry of Health website (http://www.moh.ps/attach/441.pdf, accessed 14 April 2012). A total of 21 hospitals were identified in the Palestinian West Bank, and the addresses and contact information for each were extracted and recorded; 12 were governmental hospitals and the remainder were private hospitals. According to convenience sampling techniques for descriptive studies, an 80% response rate is acceptable for interview questionnaires.

### Study tool: the questionnaire

A semi-structured questionnaire was developed for the purpose of the present study (See Additional file 1). The questionnaire was designed by the Poison Control and Drug Information Centre (PCDIC) at An-Najah National University in Palestine. It was prepared in English and accompanied by an official document explaining the purpose and importance of the survey. The questionnaire contained three sections. The first asked about the epidemiological data pertaining to the types of poisoning cases admitted. This section of the questionnaire was designed to be self-administered by ED physicians. A list of common toxic agents was compiled from the published previous studies in Palestine [3,24,25]. The participants were asked to give an ascending ranking of the exposures on this list according to their observed incidence during the last year. The agent that was ranked first (i.e. the most common exposure presenting to that hospital) during the last year was given 10 points, while the agent that was ranked last was given 1 point based on ranking order method [26]. The resulting score is a measure of the relative frequency with which a given toxic agent was observed to cause exposure compared to the other most common agents at a given ED in Palestine and across the region, a higher score reflecting an agent observed more frequently to cause exposure leading to presentation at an ED. After summation of the scores of all respondents, we obtained an aggregate ranking of the ten most common toxic agent exposures presenting to the hospitals in Palestine. The second section was designed to indicate which resources were available in their EDs, such as equipment for decontamination, life support, and elimination enhancement instruments. A list of commonly required resources used for the treatment of acute poisoning was compiled from the published recommendations [8,27,28].

The final section of the questionnaire consisted of questions to indicate which antidotes they had available in their hospital. A list of commonly required antidotes and essential drugs used for treating poisoning complications was compiled from the World Health Organization (WHO) and from published guidelines for antidote stocking [11,29]. Furthermore, the availability of antidotes in each hospital was compared to the 16 antidotes considered essential in the guidelines of Dart et al. for stocking antidotes [11].

The director of each hospital was asked permission to have the responsible participants complete the questionnaire. The heads of the ED departments were requested to complete the first section, the director of the pharmacy was requested to fill in the second section, and the third section was completed by a specialist chosen by the heads of the ED departments.

### Ethical approval

This study received approval from the PMOH and Institutional Review Board (IRB) at An-Najah National University before the initiation of the study. Verbal consent was obtained from the participants prior to commencement of the study and the requirement for written informed consent was waived. The IRB waived for protocols that were clearly below minimal risk and the research did not involve any therapeutic intervention.

### Statistical analysis

Data extracted from the questionnaires were entered into the Statistical Package for Social Sciences (SPSS version 16) software. A descriptive comparative analysis of statistical data is used. Continuous data are presented as mean ± standard deviation (SD), and categorical data are expressed as numbers with percentages. For comparative analysis, either the Chi square or the Fisher’s exact test was used to test the statistical significance of differences between categorical variables. A p value of less than 0.05 was considered statistically significant.

## Results

Eighteen hospitals returned the survey, resulting in a response rate of 85.7% (Table 1). Table 2 shows the top 10 toxic agents most frequently reported by EDs to have caused human exposure presenting at least once during the last year at their hospital. By analysing the collected data, paracetamol poisoning was ranked first (mean frequency score = 7.6 ± 2.1 points), followed by bee stings (mean = 6.9 ± 2.7 points) and organophosphates (mean = 6.7 ± 2.7 points).

**Table 1:** List of participating hospitals (18 hospitals)

**Governmental hospitals (N = 10)**	**Private hospitals (N = 8)**
Khalil Souliman (Jenin District)	Al Razi (Jenin District)
Thabet Thabet (Tulkarm District)	Al Zakah (Tulkarm District)
Al Watani (Nablus District)	Specialized Arab Hospital (Nablus District)
Rafidia (Nablus District)	Al Itihad (Nablus District)
Darwish Nazal (Qalqilya District)	Al Makassad (Jerusalem District)
Yaser Arafat (Salfit District)	Al Ahli (Hebron District)
Jericho (Jericho District)	Arab Society (Bethlehem District)
Al-Hussein (Bethlehem District)	
Princess Alia (Hebron District)	
Palestine Medical Complex (Ramallah District)	

**Table 2:** Top 10 toxic agents or classes most frequently causing exposure reported by the emergency departments during the last year (n = 18)

**Toxic agent**	**Mean score**	**Standard deviation**
Paracetamol	7.6	2.1
Bee sting	6.9	2.7
Organophosphate	6.7	2.7
Scorpion bite	6.6	2.6
Kerosen	6.1	2.4
Snake bite	5.8	2.4
Non-steroidal anti-inflammatory drugs	5.4	2.6
Chlorine	5.1	2.3
Central Nervous System medications	5.1	2.3
Cardiovascular medications	2.5	1.6

### Availability of decontamination resources

The availability of decontamination resources varied substantially according to hospital type. However, these differences did not reach statistical significance. Concerning the resources for performing decontamination through gastric lavage (GL) in the poisoned patient, nasogastric tubes were available in both types of hospitals. The availability of orogastric tubes was less than that of nasogastric tubes. According to activated charcoal (AC) dosage form, AC syrup was found to be more common than the tablet and powdered forms, and it was available in the majority of EDs in the hospitals investigated (83.3%). Furthermore, sorbitol, ipecac syrup, and polyethylene glycol were almost never available, especially in governmental hospitals (Table 3).

**Table 3:** Availability of decontamination resources stratified by hospital type (n = 18)

**Resources**	**Total**	**Private**	**Government**	**P-value**
	**N = 18 (%)**	**N = 8 (%)**	**N = 10 (%)**	
Nasogastric tube	18 (100)	8 (100)	10 (100)	>0.999
Orogastric tube	13 (72.2)	6 (75)	7 (70)	>0.999
Charcoal tablet	4 (22.2)	3 (37.5)	1 (10)	0.275
Charcoal powder	5 (27.8)	2 (25)	3 (30)	>0.999
Charcoal syrup	15 (83.3)	5 (62.5)	10 (100)	0.069
Magnesium sulphate	15 (83.3)	6 (75)	9 (90)	0.559
Sodium sulphate	11 (61.1)	6 (75)	5 (50)	0.367
Sorbitol	2 (11.1)	2 (25)	0 (0.0)	0.183
Ipecac syrup	3 (16.7)	3 (37.5)	0 (0.0)	0.069
Polyethylene glycol	1 (5.6)	1 (12.5)	0 (0.0)	0.444

### Availability of stabilisation resources

For stabilisation resources, 11 items which are useful in monitoring or treating poisoned patients were considered. Differences in availability of such resources did not reach significant difference by hospital type, as most resources were found in both types of hospital.

Six items out of the 11 were available in all 18 hospitals (100%). These items were blood pressure apparatus, IV cannula, crystalloid, nasal catheter, oxygen mask, and endotracheal tube. In addition, 3 items of the 11 were available in more than 80% of all types of hospitals. However, the availability of the remaining resources, which include colloids, and, in particular, pacemakers, was found to be less common (Table 4). However, the availability of such resources was much better compared to decontamination and elimination resources.

**Table 4:** Availability of stabilisation resources stratified by hospital type (n = 18)

**Resources**	**Total**	**Private**	**Government**	**P-value**
	**N = 18 (%)**	**N = 8 (%)**	**N = 10 (%)**	
Blood pressure apparatus	18 (100)	8 (100)	10 (100)	>0.999
IV cannula	18 (100)	8 (100)	10 (100)	>0.999
Nasal catheter	18 (100)	8 (100)	10 (100)	>0.999
Laryngeal mask airway	16 (88.9)	8 (100)	8 (80)	0.477
Oxygen mask	18 (100)	8 (100)	10 (100)	>0.999
Endotracheal tube	18 (100)	8 (100)	10 (100)	>0.999
Mechanical ventilator	16 (88.9)	7 (87.5)	9 (90)	>0.999
Colloid	13 (72.2)	7 (87.5)	6 (60)	0.314
Crystalloid	18 (100)	8 (100)	10 (100)	>0.999
Pacemaker	6 (33.3)	3 (37.5)	3 (30)	>0.999
Electrical defibrillation	17 (94.4)	7 (87.5)	10 (100)	0.444

### Availability of elimination enhancement resources

For the availability of techniques used to enhance the elimination of toxic substances, there were variations between the hospitals types. However, these differences did not reach statistical significance, except for haemodialysis (p = 0.003), where the availability of this technique was far better in governmental hospitals compared to private hospitals (90% vs. 12.5%). Haemoperfusion, haemofiltration, alkaline diuresis, acid diuresis, and peritoneal dialysis were almost never available, especially in private hospitals (Table 5).

**Table 5:** Availability of elimination enhancement resources stratified by hospital type (n = 18)

**Resources**	**Total**	**Private**	**Government**	**P-value**
	**N = 18 (%)**	**N = 8 (%)**	**N = 10 (%)**	
Haemodialysis	10 (55.6)	1 (12.5)	9 (90)	0.003
Haemoperfusion	3 (16.7)	1 (12.5)	2 (20)	>0.999
Haemofiltration	2 (11.1)	1 (12.5)	1 (10)	>0.999
Alkaline diuresis	2 (11.1)	0 (0.0)	2 (20)	0.477
Acid diuresis	2 (11.1)	0 (0.0)	2 (20)	0.477
Peritoneal dialysis	1 (5.6)	0 (0.0)	1 (10)	>0.999
Exchange transfusion	5 (27.8)	2 (25)	3 (30)	>0.999

### Availability of antidotes and essential drugs

The overall availability of each antidote in the first list varied widely; it ranged from zero (for fomepizole, cyanide kit and dimercaprol) to 100% (for atropine sulphate, calcium gluconate and sodium bicarbonate). However, four antidotes were severely deficient in hospitals (available in less than 20% of all hospitals). Those included digoxin immune Fab, polyvalent snake anti-venom, pralidoxime, and pyridoxine. None of the responding hospitals stocked all of the antidotes on the list.

In relation to hospital type, there is variability in the availability of antidotes. However, these differences did not reach statistical significance except for deferoxamine (p < 0.001). Deferoxamine was available in all governmental hospitals, but none of the private hospitals stocked it. Atropine sulphate, calcium gluconate and sodium bicarbonate were available in all governmental and private hospitals (Table 6).

**Table 6:** Availability of recommended antidotes* stratified by hospital type (n = 18)

**Antidote list**	**Total**	**Private**	**Government**	**P-value**
	**N = 18 (%)**	**N = 8 (%)**	**N = 10 (%)**	
Atropine sulphate	18 (100)	8 (100)	10 (100)	>0.999
Calcium gluconate	18 (100)	8 (100)	10 (100)	>0.999
Deferoxamine	10 (55.6)	0 (0.0)	10 (100)	0.000
Digoxin immune Fab	2 (11.1)	1 (12.5)	1 (10)	>0.999
Dimercaprol	0 (0.0)	0 (0.0)	0 (0.0)	>0.999
Ethanol (100%)	5 (27.8)	3 (37.5)	2 (20)	0.608
Fomepizole	0 (0.0)	0 (0.0)	0 (0.0)	>0.999
Glucagon	8 (44.4)	6 (75)	2 (20)	0.054
Methylene blue	7 (38.9)	2 (25)	5 (50)	0.367
N-acetylcysteine	8 (44.4)	6 (75)	2 (20)	0.054
Naloxone	17 (94.4)	8 (100)	9 (90)	>0.999
Polyvalent anti-venom	3 (16.7)	0 (0.0)	3 (30)	0.216
Pralidoxime	1 (5.6)	1 (12.5)	0 (0.0)	0.444
Pyridoxine	1 (5.6)	1 (12.5)	0 (0.0)	0.444
Sodium bicarbonate	18 (100)	8 (100)	10 (100)	>0.999
Cyanide Kit	0 (0.0)	0 (0.0)	0 (0.0)	>0.999

*According to guidelines from Dart et al. antidotes [11].

The availability of other antidotes in the second list varied widely from zero (for calcium disodium edetate) to 100% (for flumazenil and vitamin k). All hospitals stock flumazenil and vitamin k. In contrast, calcium disodium edetate was the only antidote on the list that was not available at any hospital.

By hospital type, the availability of essential drugs varied substantially. However, these differences did not reach statistical significance in all cases. Overall, the availability of most items was excellent, ranging from more than 70% to 100%, except for thiamine, isoproterenol, leucovorin, and physostigmine (Table 7).

**Table 7:** Availability of other antidotes and essential drugs in hospitals stratified by hospital type (n = 18)

**Antidote list**	**Total**	**Private**	**Government**	**P-value**
	**N = 18 (%)**	**N = 8 (%)**	**N = 10 (%)**	
**Availability of other antidotes**				
Calcium disodium edetate	0 (0.0)	0 (0.0)	0 (0.0)	>0.999
Epinephrine	14 (77.8)	6 (75)	8 (80)	>0.999
Flumazenil	18 (100)	8 (100)	10 (100)	>0.999
Isoproterenol	8 (44.4)	2 (25)	6 (60)	0.157
leucovorin	5 (27.8)	1 (12.5)	4 (40)	0.314
Protamine sulphate	17 (94.4)	7 (87.5)	10 (100)	0.444
Vitamin K	18 (100)	8 (100)	10 (100)	>0.999
Physostigmine salicylate	3 (16.7)	2 (25)	1 (10)	0.559
**Availability of essential drugs**				
Dopamine	16 (88.9)	7 (87.5)	9 (90)	>0.999
Bronchodilators	16 (88.9)	7 (87.5)	9 (90)	>0.999
Corticosteroids	15 (83.3)	7 (87.5)	8 (80)	>0.999
Antihistamines	18 (100)	8 (100)	10 (100)	>0.999
Thiamine	1 (5.6)	1 (12.5)	0 (0.0)	0.444
Dextrose	16 (88.9)	8 (100)	8 (80)	0.477
Diazepam	17 (94.4)	8 (100)	9 (90)	>0.999
Phenytoin	13 (72.2)	6 (75)	7 (70)	>0.999
Morphine	17 (94.4)	8 (100)	9 (90)	>0.999
NSAIDs	14 (77.8)	7 (87.5)	7 (70)	0.588

*Abbreviation*: NSAIDs Nonsteroidal anti-inflammatory drugs.

## Discussion

The results of this study indicate that most Palestinian hospitals have certain important immediate interventions such as gastrointestinal decontamination techniques and resources to enhance poison elimination. Currently, there are no generally recognised specific criteria that define the preparedness of an ED for the management of acute toxic exposures and poisonings. A list of commonly required resources and items which might be used for the treatment of acute poisoning was compiled from the published recommendations [8,11,16,27-31].

Unfortunately, the data of the present study showed that the resources required for performing GL, such as nasogastric and orogastric tubes, were more common than other preparations used to decrease the absorption of toxic agents such as charcoal, laxatives, and WBI. This finding is consistent with the result of a previous study of poisoning in Palestine that showed among the cases which had undergone a decontamination procedure, GL was the most commonly used [3]. Use of other decontamination resources, besides GL, which is assumed to be still commonly practiced in the surveyed hospitals of this study, was consistent with the common practices recommended in the clinical literature [32-35], which support the limited use of all types of GI decontamination of acutely poisoned patients. Decontamination of severely poisoned patients must only be undertaken after careful consideration of the potential risks and benefits of the decontamination practice [34-37].

The data of the present study also show that resources for performing decontamination through GL, such as nasogastric tubes, are available in all EDs of both hospital types (100%). Based on American Academy of Clinical Toxicology (AACT) and European Association of Poisons Centres and Clinical Toxicologists (EAPCCT) recommendations [38], there is no evidence showing that GL should be used routinely in the management of poisonings. GL should not be performed routinely, if at all, for the treatment of poisoned patients. However, the results of this study are not compatible the published recommendations, which suggest that GL is still commonly practiced in the surveyed hospitals. Serious risks of the procedure include aspiration pneumonitis, dysrhythmias, fluid and electrolyte abnormalities, hypoxia, laryngospasm, and perforation of the GI tract or pharynx [38,39].

The present study showed that ipecac syrup is not available in more than 83.3% of EDs of both type of hospitals. These findings are consistent with current recommendations that indicate that ipecac syrup should not be used routinely after poisoning exposures due to the lack of evidence of improved outcomes and risks, including reduced effectiveness of AC, delayed administration of oral antidotes, aspiration pneumonitis, and other complication of prolonged emesis [6]. Further, the study showed that AC is not available in the majority of EDs. However, charcoal syrup was available in 83.3% of the surveyed hospitals. The administration of AC is considered a useful decontamination technique if a patient has ingested a potentially toxic amount of a poison up to 1 hour previously which is known to be adsorbed by charcoal [40].

Moreover, the data of this study indicate that the resources for performing decontamination through whole bowel irrigation (WBI), such as polyethylene glycol, are not available in the majority of the EDs. Based on AACT and EAPCCT recommendations, WBI should be considered only for potentially toxic ingestions of sustained-release or enteric-coated drugs, and iron and lead toxicity, particularly for patients presenting more than 2 hours after drug ingestion and for acute drug poisoning. WBI should be considered for poisoned patients who have ingested large amounts of iron, as the levels of morbidity in these patients are high and there is a lack of other alternative techniques for GI decontamination [34].

The administration of cathartics alone has no role in the management of poisoning, and is not recommended as a method of GI decontamination [37]. Despite this, cathartics were available in the majority of EDs of the hospitals surveyed in this study. Sorbitol was rarely available, particularly in governmental hospitals. Controversy remains over the use of cathartics to hasten elimination of toxins from the gastrointestinal tract. Some toxicologists still use cathartics routinely when giving AC even though few data exist to support their efficacy [41].

The results of our study concerning the availability of GI decontamination resources are consistent with the Malaysian study performed by Awang et al. [8], except for AC dosage form. The authors, through the survey that they conducted, reported that the availability of charcoal tablets was better than powdered form, and that they were available in more than two-thirds of the EDs. In addition, the authors did not evaluate the availability of charcoal syrup in their study, which was found to be available in 83.3% of hospitals in our study.

Our present study showed that certain resources used for the stabilisation of poisoned patients who presented to EDs were commonly reported as available by Palestine hospitals. A review of the published studies indicate that supportive measures including maintenance of ABCs are frequently necessary before confirmation of intoxication [30,42,43]. Endotracheal intubation is not always necessary, but if respiratory inadequacy is present, it is better to secure the airway. Intubation is indicated in cases of acute respiratory failure. Other specific indications include the need for high levels of supplemental oxygen in carbon monoxide poisoning cases, and the need to secure the airway for gastric emptying [30]. Endotracheal intubation reduces the risk of aspiration [44], this was consistent with our findings, as the availability of endotracheal tubes was 100% in both types of hospitals. With regard to airway support resources, all hospitals had IV cannulas, nasal catheters, oxygen masks and endotracheal tubes.

Further, our findings regarding the availability of stabilisation resources were also compatible with the results of Awang et al. [8], except for volume expanders (colloids), in which their availability was much better and reached 100%.

In the present study, results showed that haemodialysis was widely available in most hospitals, which might result in an increased used of this technique to enhance the elimination of specific toxic agents. Surprisingly, among elimination enhancement resources, acid diuresis was available in 11% of Palestinian hospitals. However, acid diuresis is no longer recommended or used in poisoning treatment. It is a therapy which is associated with significant risk and little benefit, and its use has been abandoned [45]. In our study, it is unclear why the respondents indicated that alkaline diuresis is nearly unavailable, however, the data indicated that intravenous catheters, crystalloid and sodium bicarbonate were widely available. This may be because the respondents are unfamiliar with the use of these agents in some poisoning cases treatment, or the therapy is unavailable for some reasons, e.g. inability to check blood gases due to unavailability of arterial blood gas analyzer. It is clear that the majority of governmental hospitals (e.g. haemodialysis resources) perform some elimination enhancement techniques as they have the proper facilities for that, whereas most of the private hospitals do not perform them due to the lack such of facilities. There were no apparent differences in the availability of elimination enhancement resources between Palestine and Malaysia, except for peritoneal dialysis, which was available in 51.4% of the Malaysian hospitals and was considered one of the most common techniques used to enhance the elimination of toxic substances [8].

Our results show that a large percentage of antidotes are not available in the surveyed hospitals. Certain important antidotes, which are included in the essential drugs list implemented by the PMOH, are not stocked by a substantial number of hospitals, including governmental hospitals. Examples of such antidotes include pralidoxime for organophosphate poisoning and calcium disodium edetate for heavy metal poisoning. The World Health Organization (WHO) documented a serious shortage in supplies of essential drugs and disposables reported by the PMOH in Palestine. PMOH reported that 101 drugs (19% of 523 drugs on the essential drug list) and 61 medical disposables items (8% of 720 essential items) were exhausted [46].

Among the hospitals, paracetamol toxic exposure was the most frequently reported case by EDs, followed by bee stings and organophosphate exposure. Surprisingly, only two governmental hospitals had the antidote for paracetamol poisoning (N-acetylcysteine) in stock. The current study showed that few hospitals hold antidotes for digoxin toxicity and isoniazid poisoning, which is in keeping with findings reported from previous studies that these antidotes are rarely requested [3,24]. In addition, the availability of an antidote to treat individual patients who have been poisoned with cyanide was inadequate, as the 18 hospitals held no antidote. Furthermore, the availability of fomepizole, which is used as an antidote for ethylene glycol and methanol toxicity, was the same as that of the cyanide kit. As previously reported by Al-Sohaim et al. [16] and Sawalha et al. [20], antidotes used to treat conditions other than poisoning and toxic drug exposure were more frequently stocked. Atropine sulphate, calcium glyconate, dopamine, diazepam and sodium bicarbonate were available in the majority of hospitals of both types.

Allergic reaction to bee stings is like other allergic reactions. Mild reactions are treated with an antihistamine such as diphenhydramine. If a more severe reaction develops, epinephrine should be administered [47]. Epinephrine was available in 77.8% of the surveyed hospitals. This was also important, since anaphylaxis/allergic reaction and serum sickness have been reported after the administration of anti-venom for snake bites [10].

Our study is also the first in Palestine to assess the antidotes stocked at a national level. Insufficient antidote stocking is not a unique problem to Palestine. Our findings are also consistent with studies from multiple countries which report variable and inadequate antidote stocking levels. A recent study carried out by Al-Sohaim et al. [16] found that no hospital had sufficient stock of 16 antidotes. Wium and Hoffman [48] conducted a study in South Africa. The results of the study revealed that there was a problem with regard to the availability and distribution of important antidotes, as none of the responding hospitals stocked all of the antidotes on the list. A similar study performed in north Palestine that was carried out by Sawalha et al. [20] showed that the number of antidotes stocked in all hospitals ranged from 5 to 12, but that no hospital stocked all 25 of the antidotes listed. An Australian study carried out by Nissen et al. [15] surveyed Queensland hospitals as to the level of stocks held of 13 antidotes. This study reported that while most hospitals stocked some important antidotes, no hospital stocked all 13 and few hospitals had sufficient stocks to treat an adult patient.

There has been no study exploring the reasons for inadequate antidote stocking in Palestine. Abbott et al. [9] suggested some possible reasons for inadequate stocking of antidote in their study in New Zealand. The extreme rarity of needing the antidote due to the low frequencies of poisonings was the most common reason for the low availability of antidotes. High costs, short shelf-lives, having agents that might have benefits in poisoning management and a lack of clinical requests were other contributory factors for inadequate stocking in the New Zealand hospital pharmacies [9].

### Strengths and limitations

The major strength of the current study is that it is the first of its kind to assess the level of readiness of hospitals for the management of acute toxic exposures and poisoning in Palestine. Furthermore, ours is also the first study to assess the antidotes stock level throughout Palestine, except for Gaza, due to the lack of access and mobility. There was a previous study carried out by Sawalha et al. [20], where the authors conducted a survey that screened the stocking of specific antidotes at hospitals in the north of Palestine only.

This study is subject to a number of limitations. The objectives of the study were only to document the availability of immediate interventions (gastrointestinal decontamination techniques, patient stabilization resources and methods to enhance elimination) and the availability of antidotes and to evaluate the impact of hospital types on the availability of these resources for the management of acute toxic exposure and poisonings in Palestinian hospitals. We did not study other factors that may determine the appropriateness of these resources and whether those resources are specifically used for poisoning or for other indications. Also, we have not performed a study that clarifies the demographic, aetiological and clinical characteristics of actual poisoning cases, as some poisoning cases that occur in the northern districts may differ from those that occur in the southern districts; therefore, we may find variety in the availability of antidotes between districts. Furthermore, these study findings are based entirely on a self-administered questionnaire survey. The data collected depend upon the knowledge and responsiveness of the respondents, which carries inherent risks of reporting error or bias. Thus, the results might not reveal the current levels of readiness and antidote stocks in Palestinian hospitals.

## Conclusions and recommendations

This study looked at 18 hospitals. The availability of treatment resources and antidotes in Palestinian hospitals were not adequate, except for stabilisation resources. According to hospital type, there was variability in the availability of these resources, particularly in the availability of resources that enhance the elimination of poison. Most Palestinian hospitals stocked some of the surveyed antidotes. In relation to hospital type, there was great variability in the availability of antidotes. The availability of most antidotes was far better in government hospitals compared to private hospitals. There are no guidelines in place as to which antidotes should be considered essential for use in Palestine, so specific antidote stocking guidelines might be required and useful in Palestinian hospitals. Coordination between PCDIC and hospitals should be established regarding emergency facilities for the effective management of poisoning cases, as well as the type and quantity of antidotes in each hospital in order to direct the poisoned patients to the hospital where the appropriate management resources and suitable antidote are available. Since this study is descriptive in nature, it serves as baseline data for further studies related to acute toxic exposure and poisoning. In addition, we need additional studies evaluating quantities of antidotes, the clinical use of antidotes, the factors that affect the availability of antidotes, the types of poisoning cases seen, diagnostic equipment that would help treating the undifferentiated poisoned patients, and the quality of treatments provided to such cases at various hospitals throughout Palestine.

## Abbreviations

IRB: Institutional review board; SPSS: Statistical package for social sciences; SD: Standard deviation; GI: Gastrointestinal; EDs: Emergency departments; ABC: Airway, breathing and circulation; PCDIC: Poison control and drug information centre; WHO: World Health Organization; PMOH: Palestinian ministry of health; GL: Gastric lavage; AC: Activated charcoal; AACT: The American academy of clinical toxicology; EAPCCT: European association of poisons centres and clinical toxicologists; WBI: Whole bowel irrigation; UNRWA: the United Nations relief and works agency for Palestine refugees in the near east; NGOs: Non-governmental organizations; PMMS: Palestinian military medical services.

## Competing interests

The authors declare that they have no competing interests.

## Authors’ contributions

All authors were involved in drafting the article, and all authors approved the final version to be submitted for publication. SZ conceived of the study conception and design, organized and supervised the data collection, and provided analysis, interpretation, and writing. SA and WS participated in the study design, and provided critical revision of manuscript for important intellectual content. YB, and AA contributed to the data collection, results tabulation and writing. RA was involved in concept and editing the manuscript.

## Authors’ information

Dr. Sa’ed Zyoud is an assistant professor of clinical toxicology/pharmacy and the head of a research group in the field of clinical toxicology, clinical pharmacology/pharmacy, social pharmacy, pharmacoepidemiology and drug safety, and a bibliometrics analysis. S.Z and the research group have published many articles in leading international and high reputation journals. The research group has also supervised many students in the fields of nursing, public health and pharmacy.

## Supplementary Material

Additional file 1 The questionnaire. Click here for file
