# Supplementary material for: Availability of treatment resources for the management of acute toxic exposures and poisonings in emergency departments among various types of hospitals in Palestine: a cross-sectional study
Source: Scand J Trauma Resusc Emerg Med. 2014 Feb 21;22:13. doi: 10.1186/1757-7241-22-13 (PMC3939642; doi:10.1186/1757-7241-22-13)
Supplement: Additional file 1 — The questionnaire. [file 1757-7241-22-13-S1.docx]

***Additional file 1: The questionnaire***

**This survey is carried out by researchers at An-Najah National University for the purposes of scientific research for evaluating the availability of treatment resources for the management of acute toxic exposures and poisonings in emergency departments among various types of hospitals in Palestine.**

**Please kindly answer all questions carefully, noting that it will be used for the purposes of scientific research.**

**===================================================================**

***First Section***

Please indicate the following:

*** Date:** ……………..

*** Hospital Name:** ……………..

*** Type of hospital:** ……………..

*** City:** ……………..

*** Does the hospital receive cases of poisoning:** □ Yes □ No

======================================================

*** Rank ascendingly the most frequent 10 toxic agents existed in the list according to your observations during the last year.**

| **Toxic agent** | **Rank** | **Toxic agent** |
| --- | --- | --- |
| Paracetamol |  |  |
| Kerosen |  |  |
| Snake Bite |  |  |
| Non-steroidal anti-inflammatory drugs |  |  |
| Bee Sting |  |  |
| Organophosphate |  |  |
| Scorpion Bite |  |  |
| Chlorine |  |  |
| Central Nervous System medications |  |  |
| Cardiovascular medications |  |  |
| Others ( specify)………. |  |  |

***Second Section***

Please indicate the following:

*** Date:** ……………..

*** Hospital Name:** ……………..

*** Type of Hospital:** ……………..

*** City:** ……………..

*** Department Supervisor (Choose only one):**

□ Doctor □ Pharmacist □ Nurse □ Other

*** Does the hospital receive cases of poisoning:** □ Yes □ No

======================================================

*** Put X if the decontamination resources are available or not in the hospital:**

| **No** | **Yes** | **Resources** |
| --- | --- | --- |
|  |  | Nasogastric tube |
|  |  | Orogastric tube |
|  |  | Charcoal tablet |
|  |  | Charcoal powder |
|  |  | Charcoal syrup |
|  |  | Magnesium sulphate |
|  |  | Sodium sulphate |
|  |  | Sorbitol |
|  |  | Ipecac syrup |
|  |  | Polyethylene glycol |

*** Put X if the stabilization resources are available or not in the hospital:**

| **Resources** | **Yes** | **No** |
| --- | --- | --- |
| Blood pressure apparatus |  |  |
| IV cannula |  |  |
| Nasal catheter |  |  |
| Laryngeal mask airway |  |  |
| Oxygen mask |  |  |
| Endotracheal tube |  |  |
| Mechanical ventilator |  |  |
| Colloid  Hydroxyethyl starch  Gelofusine |  |  |
| Crystalloid  Normal saline  Lactated Ringer's solution  Glucose (dextrose) |  |  |
| Pacemaker |  |  |
| Electrical defibrillation |  |  |

*** Put X if the enhancement resources are available or not in the hospital:**

| **Resources** | **Yes** | **No** |
| --- | --- | --- |
| Haemodialysis |  |  |
| Haemoperfusion |  |  |
| Haemofiltration |  |  |
| Alkaline diuresis |  |  |
| Acid diuresis |  |  |
| Peritoneal dialysis |  |  |
| Exchange transfusion |  |  |

***Third Section***

Please indicate the following:

*** Date:**……………..

*** Hospital Name:**……………..

*** Type of hospital:**……………..

*** City:**……………..

*** Does the hospital receive cases of poisoning:** □ Yes □ No

**========================================================**

*** Put X if these antidotes are available or not in the hospital:**

| **Antidote list** | **Yes** | **No** |
| --- | --- | --- |
| Atropine sulphate |  |  |
| Calcium gluconate |  |  |
| Deferoxamine |  |  |
| Digoxin immune Fab |  |  |
| Dimercaprol |  |  |
| Ethanol (100%) |  |  |
| Fomepizole |  |  |
| Glucagon |  |  |
| Methylene blue |  |  |
| N-acetylcysteine |  |  |
| Naloxone |  |  |
| Polyvalent anti-venom |  |  |
| Pralidoxime |  |  |
| Pyridoxine |  |  |
| Sodium bicarbonate |  |  |
| Cyanide Kit |  |  |

**=======================================================**

*** Put X if the other antidotes and essential drugs are available or not in the hospital:**

| **Antidote list** | **Yes** | **No** |
| --- | --- | --- |
| **Availability of other antidotes** | | |
| Calcium disodium edetate |  |  |
| Epinephrine |  |  |
| Flumazenil |  |  |
| Isoproterenol |  |  |
| Leucovorrin |  |  |
| Protamine sulphate |  |  |
| Vitamin K |  |  |
| Physostigmine salicylate |  |  |
| **Availability of essential drugs** | | |
| Dopamine |  |  |
| Bronchodilators |  |  |
| Corticosteroid |  |  |
| Antihistamine |  |  |
| Thiamine |  |  |
| Dextrose |  |  |
| Diazepam |  |  |
| Phenytoin |  |  |
| Morphine |  |  |
| NSAIDs |  |  |

**Thank you for help!!**
